# The emotion bias of health product consumers in the context of COVID-19

**DOI:** 10.1371/journal.pone.0278219

**Published:** 2022-11-28

**Authors:** Lian Yuan, Mingyan Wang

**Affiliations:** School of Management, Shanghai University of Engineering Science, Songjiang, Shanghai, China; The University of the West Indies, TRINIDAD AND TOBAGO

## Abstract

The ongoing COVID-19 has led to an increase in negative emotions and health awareness among consumers. This paper discusses the emotion bias of Chinese consumers during the three periods: the pre-COVID-19 period, the COVID-19 lockdown period, and the COVID-19 normalization period. This study takes health products as the research object and crawls relevant reviews on the JD platform to classify products. The data were classified into emotion, the intensity of emotion was calculated, and the logistic regression model and variance analysis were used to analyze the difference in emotion expression. The study reveals that consumers are willing to express fear and sadness during the COVID-19 lockdown era and are willing to express like emotions before the pandemic compared to the three periods. There are also differences in the emotional intensity of different product reviews. The intensity of emotional expression is more vigorous for consumers who purchase nutritional products, while for those who purchase healthcare equipment, the intensity of emotional expression is lower. This study offers the emotion bias of consumers in response to COVID-19 to provide a theoretical basis and reference solution for implementing marketing strategies for health product companies.

## 1 Introduction

Coronavirus (CoV) is derived from the Latin word "cor-ona", meaning crown [1] and was first discovered in the 1960s [2]. COVID-19 was first identified in Wuhan, China, in late 2019 and spread rapidly worldwide in the following months. The first case of COVID-19 was reported in a Caribbean island country on 2020/3/10, three months later than in China [3]. At this time, about 3,000 new COVID-19 confirmed cases were being reported daily in European countries such as Italy, and the cumulative number of COVID-19 confirmed cases rapidly exceeded 10,000. COVID-19 was cleared in China on 8 April 2020, at a time when the global COVID-19 epidemic was severe. China is still adhering to the policy approach of dynamic zero, while other countries have declared coexistence with COVID-19.

On 11 March 2020, the World Health Organization (WHO) proclaimed the outbreak of coronavirus disease 2019 (COVID-19) a pandemic [4]. The COVID-19 pandemic has altered not only people’s emotions but also their consumption patterns [5], social behavior [6], and hygiene behaviors [7]. Several voices warned of their disruptive impact on food consumption [8]. There is little doubt that COVID-19 has had a profound impact on the livelihoods of people all across the world. The only long-term solution to combat the COVID-19 epidemic is vaccination with the COVID-19 vaccine [9]. It is also the only way to return to everyday life.

COVID-19 was widely regarded as a considerable hazard to human health, disrupting people’s lives by influencing their everyday behavior and producing emotions of fear, worry, and despair [10, 11]. COVID-19 has undoubtedly contributed to the current dread, anxiety, and uncertainty climate. When people are in negative emotional states for a long time, their physical and mental health will be severely affected [12, 13]. This undoubtedly constitutes an adverse change in consumers’ emotions compared to the pre-COVID-19 situation.

In previous studies, it has been demonstrated that when a coronavirus threatens individuals, they experience fear-related emotions. They engage in more protective behaviors and seek more relevant health-related information [14, 15]. Health-literate people are more likely to take measures to prevent the spread of COVID-19 [9]. Our views are compatible with fear appeal theories, which claim that people are motivated to undertake behavioral adjustments and learn more about health knowledge when they fear a disease’s dangers [16]. The promotion of health awareness increases the demand for health products. Consumers’ health demands have been demonstrated to influence their purchasing intentions and preferences for health products [17]. Health-conscious consumers are actively working to improve their health behaviors and healthy diet.

The healthcare sector comprises the entirety of the healthy chain, and its new mission is to improve people’s health literacy and their consumption of suitable amounts of health products. Besides, it has blossomed into a burgeoning industry with enormous market potential. Globally and in China, consumption beneficial to one’s health has become a prominent consumer trend. Medical products, health care products, nutritional products, and healthcare management are all examples of conventional health products [17]. Faced with COVID-19, the Chinese population is creating a healthy lifestyle and vigorously searching the Internet for health-related products and professional medical services. In this article, the concept of health products refers to the research of scholars such as Wang et al., including traditional Chinese and Western medicines (over-the-counter medicines), health therapy, health care underwear and weight loss, nutritional products, functional foods and so on.

During a pandemic, consumers’ emotional analysis is an important research issue. Most researchers analyzed user sentiment using particular phrases in messages [18]. Enterprises can determine a product’s popularity by understanding customers’ sentiments [19]. So far, most studies have investigated users’ sentiments on social media, like Twitter [20, 21]., and Weibo [22]. Only a few research have been investigated to explore emotional differences during crises [23, 24]. This paper is focused on the difference in consumer emotion based on health products review in JD mall during the pre-COVID-19, COVID-19 lockdown, and normalization period. To our knowledge, no studies have investigated consumer emotion from reviews of health products. There is also no empirical study to date that has considered a comparative analysis of differences in consumer emotional responses in China’s health product industry over the three periods. This study aims to fill this research gap to understand consumers’ emotion distribution and quantify consumers’ emotion scores, followed by exploiting statistical methods of logistic regression and Multivariate analysis of variance to discover differences between emotional expression and emotional intensity. If there is a discernible difference during the three periods, it may help businesses explore consumers’ purchase behavior. We hope that by doing so, we will be able to address the following three research questions:

Question 1: What are the differences between different time and product types in emotion word illustrations?
Question 2: What are the differences between different time and product types in the emotional expression of product reviews?
Question 3: What are the differences between different time and product types in the emotional intensity of product reviews?

Here’s the paper’s structure. Section 2 introduces related works about emotion analysis and the influence factor of emotion expression. Section 3 explains the emotion evaluation method and calculation method of emotional intensity. Section 4 presents the differences in emotional expression and emotional intensity. The paper comes to a close with Section 5.

## 2 Literature review

### 2.1 Consumer emotion during COVID-19

The COVID-19 coronavirus pandemic has become a global crisis. The virulence of the disease has shaken the world. Big Commerce reports that the COVID-19 pandemic has dramatically changed what, how and when people buy [25]. Unprecedented panic purchase by consumers has been confirmed by the research of Laato et al. [26]. Scholars and journalists have pointed out consumers’ stockpiling behavior during COVID-19 is a form of panic purchase [12]. In the context of the COVID-19 pandemic, supply chain disruptions resulting in empty shelves may increase consumers’ propensity to hoard [27]. Consumers over-buy certain necessities (e.g. food, water, hygiene products) [13] and reduce the consumption of unhealthy food such as unhealthy snacks, candy, and biscuits [28]. Studies have also shown that consumers’ willingness to consume healthy food has increased [11].

This world has experienced several health crises, such as SARS, Ebola, H1N1, etc. [29]. These crises impacted the world economy and health care, sparking fear, panic and anxiety in billions of people. COVID-19 affects consumer sentiment, which also means it has an impact on consumer happiness and achievement [30]. Human emotions are divided into two categories: positive and negative.

During the pandemic, positive emotions have declined, while negative emotions such as anxiety and fear have risen sharply [18]. Research shows that consumers are beginning to worry about their personal and family health and whether existing basic needs can be met [31]. The continued increase in negative consumer sentiment is socially and economically damaging and devastatingly affects the individual immune system [32].

In the last 20 years, sentiment analysis has been studied in numerous industries and with diverse methodologies [33]. Approaches that are based on emotion dictionaries and machine learning are the two primary categories of research ideas that have been proposed for emotion classification methods. The emotion dictionary-based research approach is an unsupervised classification method. It divides emotions into multiple types for research. The most extensively used English dictionary is WordNet [34], the Chinese emotional vocabulary ontology database of the Dalian University of Technology [35], and the NTUSD (Chinese sentiment Dictionary) of National Taiwan University [36] are among the Chinese open-source sentiment dictionaries. Yu et al. [37] adopted a sentiment dictionary approach to perform sentiment analysis on posts in Wuhan during the COVID-19 pandemic.

Machine-learning-based algorithms are essentially text categorization. It utilizes techniques for machine learning to classify text into three categories: positive emotion, negative emotion, and neutral emotion. Support Vector Machine (SVM) [38], Naive Bayes (NB) [39], and Maximum Entropy (ME) [40] are some of the mature classification methods. Samuel et al. [41] measured public sentiment during the pandemic using two basic machine-learning methods.

To properly divide sentiment into several types, the DUTIR Emotion Ontology set was established and maintained by Professor Lin Hongfei and his team at Dalian University of Technology Institute of Information Retrieval (DUTIR) [42], was used as the emotion lexical resource in this study. There are seven sentiment types—sadness, happiness, anger, disgust, surprise, like, or fear. The impact of negative words and degree adverbs on emotional intensity is also considered when calculating the emotional intensity of each type of emotion. We quoted negative vocabulary and degree vocabulary and assigned a different weight to calculate emotional intensity.

### 2.2 Influencing factors of emotional expression

According to existing research, consumer sentiment is mainly affected by social factors, individual factors, and product factors. Social factors mainly include the social environment and mass media. As far as the social environment is concerned, the current society is in the COVID-19 period. According to a study, unpleasant emotions like anger, fear, and disgust altered dramatically, although sadness and joy did not [43]. As far as individual factors are concerned, the current research on gender differences is abundant. Many Scholar’s research [44, 45] have shown that women were more open in expressing their emotions than men. Besides, individual emotions are also affected by age and education level. Boshoff’s research showed that as age increased, emotional expression behavior would decrease [46]. From the perspective of education level, Acevedo demonstrated that those with lower levels of education have stronger emotional expression skills than people with higher levels of education in terms of education level [47]. Regarding product factors, product categories influence consumers’ emotions when shopping offline or online. An analysis of online evaluations found a correlation between product features and consumers’ sentiment orientations [48].

In addition to existing research, product types also play an essential role in emotional expression. Each consumer’s awareness and attention to the product are different, so their response to the characteristics of different products is also inconsistent. Zhang et al. found that different product categories moderate the impact of emotions [49]. Contextual elements such as product kind influence a technique’s capacity to convey the natural feeling of a review [50].

Current research has verified that the product types have an impact on consumer emotion and have not elaborated on the emotion bias of this factor. Based on previous studies, this research mainly studies the differences between emotion type and emotion intensity and conducts a fine-grained emotion classification of health product reviews.

## 3 Method

### 3.1 Data collection and the corpus

To investigate the emotion bias of consumers in different phases, three product review datasets were constructed by crawling health product reviews from the JD Mall platform in China. The datasets contained health product reviews collected before COVID-19, during the lockdown, and during the normalization of COVID-19 prevention and control in a total of about 38000 reviews.

Our first dataset consisted of health product reviews before the pandemic. The period pre-COVID-19 was about three months between 10 November 2019 and 22 January 2020. The second dataset we used contained reviews collected during the COVID-19 lockdown in Wuhan. The period of COVID-19 was the lockdown era in Wuhan between 23 February and 8 April 2020. The third dataset we used contained reviews collected after the COVID-19 lockdown in Wuhan. The COVID-19 normalization period lasted about three months between 8 April 2020 and 1 July 2020.

**Fig 1:**
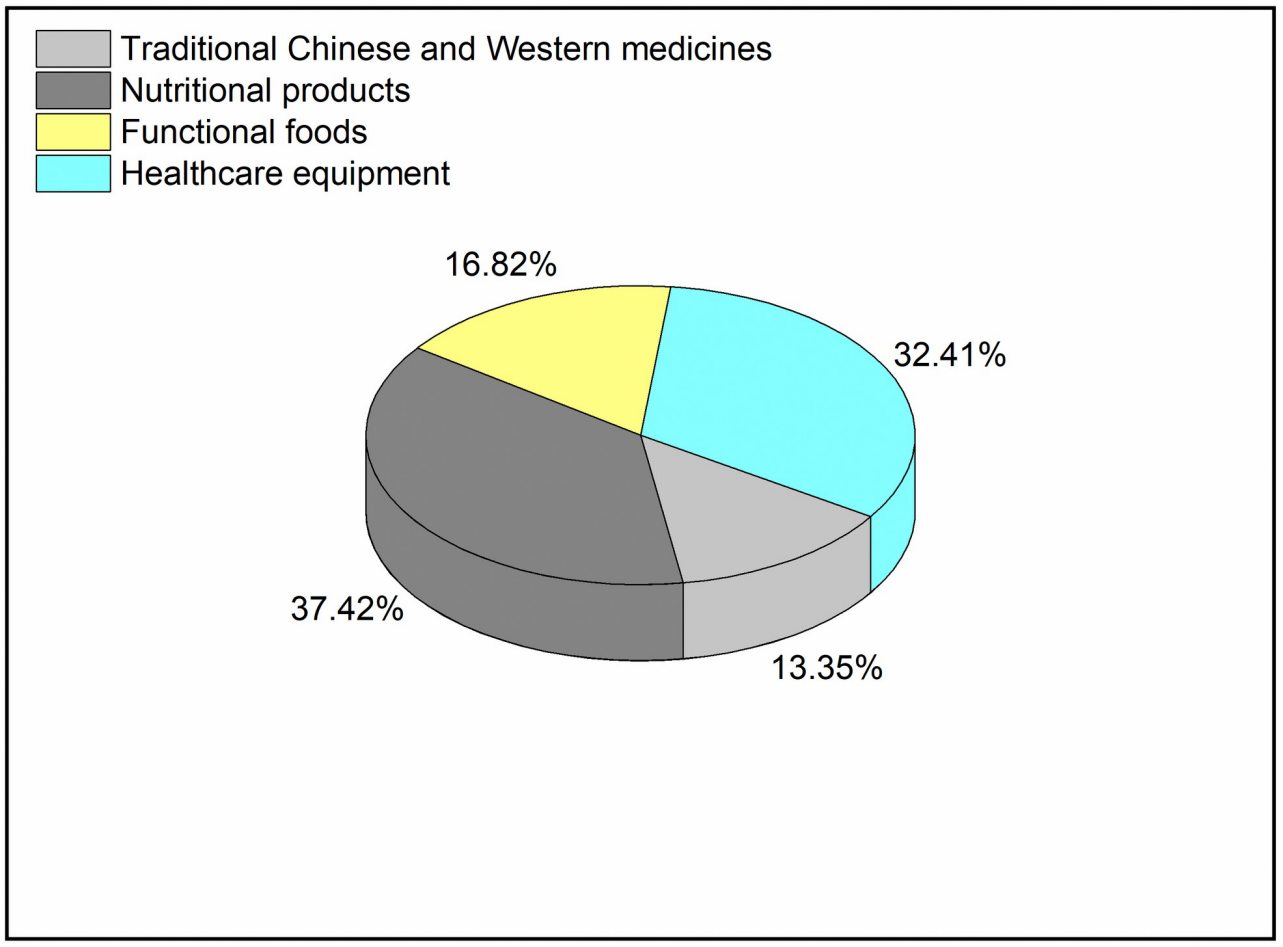
Product categories distribution.

Data were collected using a three-step procedure. The initial step was identifying health product items in the JD Mall platform. According to the broad definition of health products and JD’s product classification, we select the following four types of health products as the objects of this study: traditional Chinese and Western medicines, nutritional products, functional foods, and healthcare equipment. The second step was to filter out each of the four health products with the most reviews. For this purpose, product items with less than 5000 reviews were removed from this analysis. Those with less than 5000 reviews could be recently launched and cannot afford a thorough analysis. The third step was to crawl product reviews via crawler. The following data items were crawled: review content, and review time. Of all the entries collected, duplicated records were eliminated. As shown in Fig 1, The final dataset for analysis consisted of the remaining 37997 reviews (traditional Chinese and Western medicines = 5074, nutritional products = 14219, functional foods = 6390, healthcare equipment = 12314). And the collection and analysis method complied with the terms and conditions for the source of the data.

### 3.2 Degree adverb dictionary

A degree adverb dictionary is being compiled by us right now, and its foundation is the degree adverbs that HowNet provides. There are 219 adverbs in total. Amplifiers (such as “very”) that boost the semantic intensity of a surrounding lexical item and downtowners (such as “slightly”) that lower it is the most frequently noted in extant studies.

Every degree adverb in the lexicon is given a certain modification weight determined by whether or not it has a reinforcing or deactivating impact. More specifically, the modification weight of a degree adverb is set to 3 if it can heighten the emotional intensity of sentiment words it modifies. Examples of such degree adverbs include " extremely," "too much," and "very." The modification weight is set to 0.5 if it lessens the sentiment intensity of a sentiment term, like "little," "a little," or "a little bit."

### 3.3 Negative adverb dictionary

The negative word carries a negative connotation. When an emotionally charged term is modified with a word with a negative connotation, the initial emotional inclination will be inverted. As a result, it has an impact on the emotional polarity of the entire evaluation. For example, in Chinese, "I don’t like this apple", in this statement, the whole sentence shows a negative tendency.

If an emotive word has more than one negative adverb in front of it, the polarity is determined by the number of negative adverbs. When the number is even, the original emotional polarity is preserved; when the number is odd, the emotional polarity is transformed into an opposite site. As a result, the negative adverb’s influence factor for the emotion term *w* is described as follows:

$$w={\left(-1\right)}^{r}$$ (1)

*r* represents the number of negative adverbs.

### 3.4 Variable operationalization

In the research, product categories and time are independent variables. Time includes three periods (the pre-COVID-19 period, the COVID-19 lockdown period, and the normalization period). Product categories include traditional Chinese and Western medicines, functional foods, nutritional products, and healthcare equipment.

Here are two dependent variables. One is multiple emotions expressed in health product reviews. The other one is the emotional intensity of health product reviews. This investigation utilized the DUTIR Emotion Ontology set as the emotion lexicon resource. Then refer to dutir’s classification method to divide emotions into seven categories. Then, each review was tagged with an emotion type—sadness, happiness, anger, disgust, surprise, like, or fear. Finally, calculate the emotional intensity. The specific technique of calculation is outlined in the following:

Use *p* to represent a health product review and identify each emotional word after using the jieba tool to segment reviews, and the negative words and degree words before the emotional words. Since emotions are divided into seven categories in the emotion dictionary, when calculating the emotion of a comment, you need to calculate the emotional intensity of each emotion. For the *i* − *th* emotion, the *j* − *th* emotion word, the calculation rules of emotional intensity are as follows:

$$p_{ij}=\text{w}a_{ij}e_{ij}(1\leq i\leq 7;j>0;n\geq 0)$$ (2)

Where *a*_*ij*_ means the weight of the degree adverb, *e*_*ij*_ represents the emotional intensity. Then comprehensively calculate the emotional intensity of a certain emotion. In other words, add up the emotional intensity of all emotional words belonging to that emotion, and the formula is as follows:

$$p_{i}=\Sigma_{j=1}^{m}p_{ij}(1\leq i\leq 7;0<j<m;\;n\geq 0)$$ (3)

Where *m* represents the number of emotion words of the i-th emotion.

## 4 Results and discussion

### 4.1 Descriptive statistical analysis

The comment variable information is listed in Table 1.

**Table 1:** Product reviews’ variable information.

Variable		Number	Percentage
Time	The pre-COVID-19 period	8254	21.70%
	The COVID-19 lockdown period	14046	37.00%
	The COVID-19 normalization period	15697	41.30%
Product categories	Traditional Chinese and Western medicines	5074	13.35%
	Nutritional products	14219	37.42%
	Functional foods	6390	16.81%
	Healthcare equipment	12314	32.41%
Emotion	Like	57695	75.70%
	Happiness	9108	11.90%
	Sadness	1248	1.60%
	Anger	155	0.20%
	Fear	627	0.80%
	Disgust	7097	9.30%
	Surprise	279	0.30%

According to statistics, among the approximately 200,000 comments crawled, we screened out 8254 reviews before the epidemic (accounting for 21.7%), 14046 reviews during the epidemic lockdown period (accounting for 37.0%), and 15697 reviews during the normalization of COVID-19 prevention and control (accounting for 41.3%) for the experiment.

Table 1 reveals that like emotion had the highest proportion in the distribution of emotion words, accounting for more than 70%. And happiness emotion accounted for a proportion reaching 11.9%. Disgust emotion accounted for more than 9.3%, while the other emotions accounted for a smaller percentage of the total. Reviews related to nutritional products accounted for 37.42%, while reviews about health care equipment accounted for 32.41%. The reviews of the other two products accounted for 16.81% and 13.35%, respectively, a difference of 3.5%.

### 4.2 Emotional high-frequency words

This analysis presents a bar chart that provides excellent detail with the exact frequency of emotional words. This section compared the high-frequency words for the seven emotions at the three stages: the pre-COVID-19 period, the COVID-19 lockdown period, and the COVID-19 normalization period.

**Fig 2:**
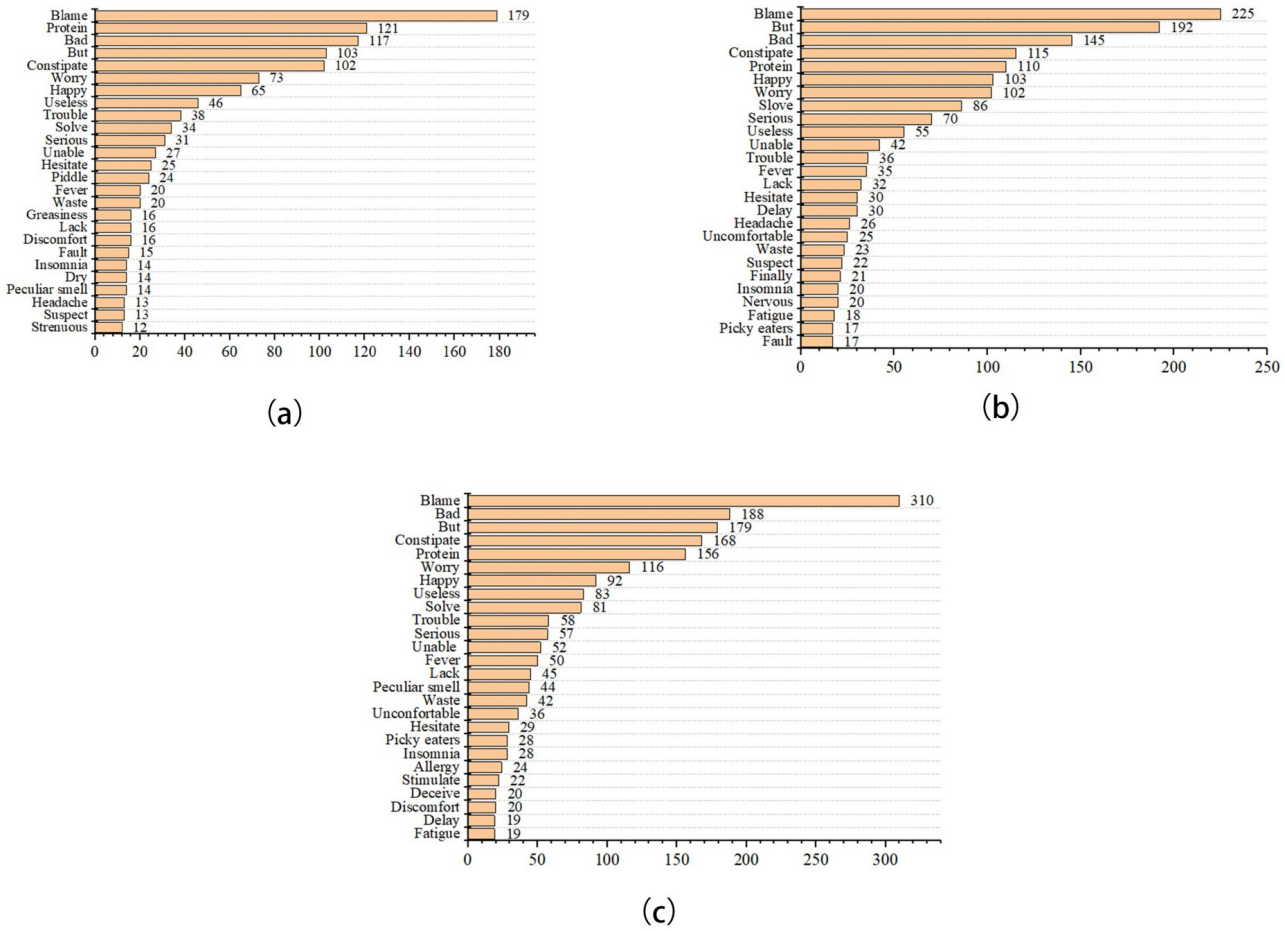
Emotional high-frequency words of disgust in the pre-COVID-19 (a), COVID-19 lockdown (b), and normalization era (c).

The results in Fig 2 showed that the most frequently appearing words were “blame”, “bad”, “but”, “constipate”, and “protein”. Comparing the three graphs above, “blame”, “bad”, and “constipate” had shown a rise. The word blame usage had more than tripled. During the COVID-19 lockdown, "protein" fell compared to before COVID-19, but it rose during COVID-19 normalization.

**Fig 3:**
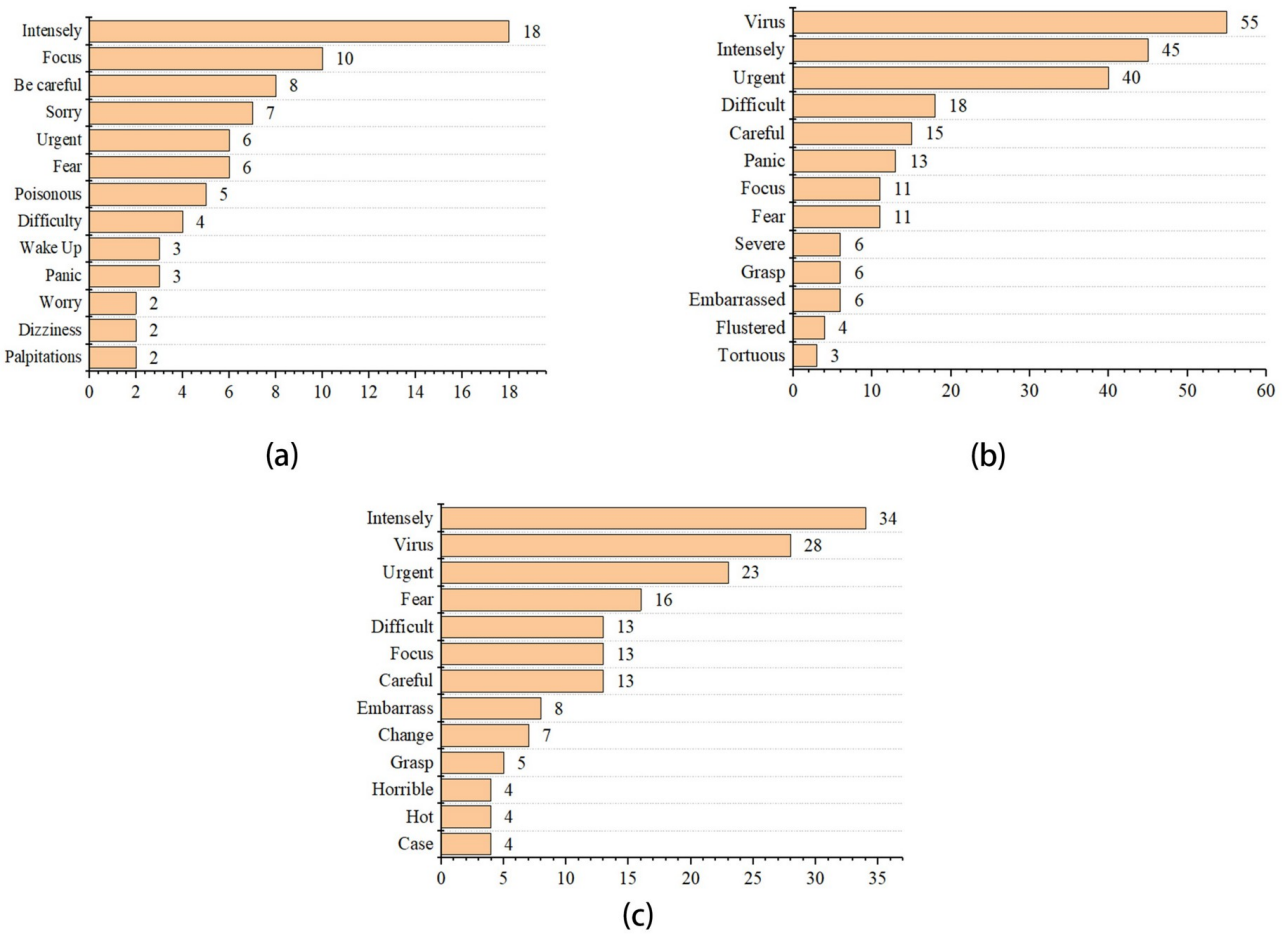
Emotional high-frequency words of fear in the pre-COVID-19 (a), COVID-19 lockdown (b), and normalization era (c).

Of the fear emotion, “intensely” was the most frequently used word related to the emotion of fear in the pre-COVID-19 period, followed by “focus”, “careful”, and “embarrassed”. During the lockdown of COVID-19, “virus” was the most used word, followed by “intensely”, “urgent”, and “difficult”. Top words that appeared in this period included “virus”, “urgent”, and “fear” during the COVID-19 normalization period. As illustrated in Fig 3, “intensely” and “virus” increased by several times during the COVID-19 lockdown and normalization period.

**Fig 4:**
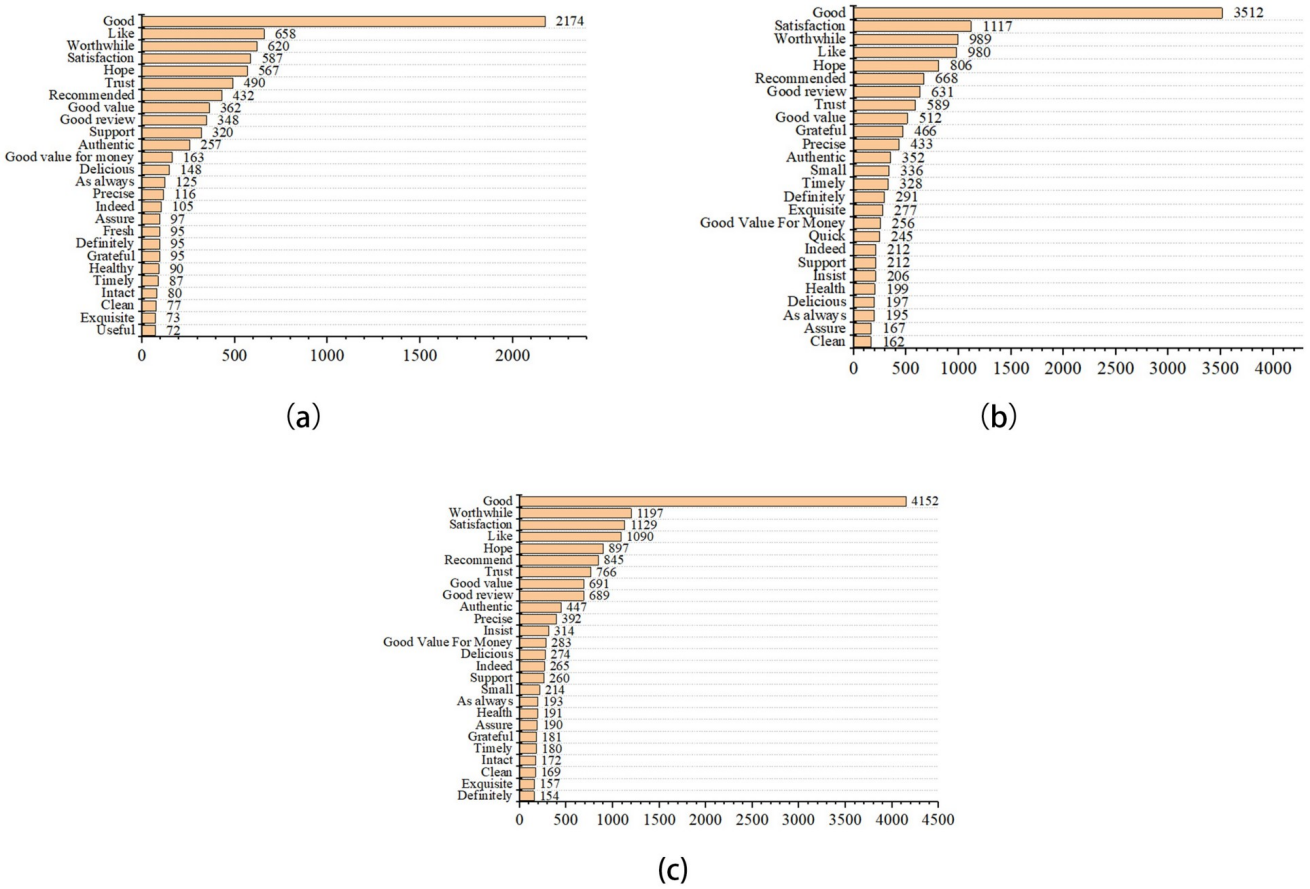
Emotional high-frequency words of like in the pre-COVID-19 (a), COVID-19 lockdown (b), and normalization era (c).

Fig 4 showed that “good”, “satisfaction”, “worth”, “like”, and “hope” were used the maximum number of times in three periods. “Exquisite”, “timely”, and “grateful” had also shown a striking rise as compared to before COVID-19. Compared to the COVID-19 normalization period, “good”, “word”, and “satisfaction” doubled before COVID-19.

**Fig 5:**
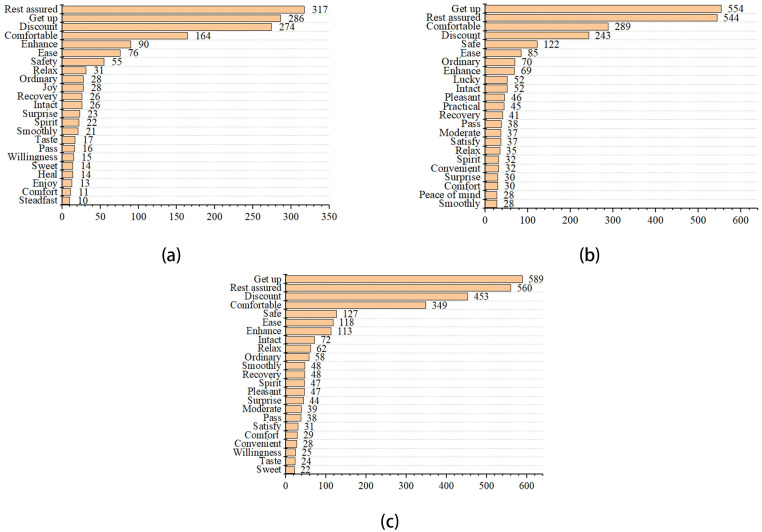
Emotional high-frequency words of happiness in the pre-COVID-19 (a), COVID-19 lockdown (b), and normalization era (c).

According to Fig 5, the most frequently appearing word was “rest assured”, and the secondary word was “stand up” in pre-COVID-19. However, the most frequently appearing word was “stand up”, followed by “rest assured” in two other phases. Besides, “safe” has also been expressed more times after the outbreak of COVID-19. “Rest assured” and “get up” nearly increased by two times during COVID-19 normalization compared to before the COVID-19 period but minimally increased during the transition from the COVID-19 lockdown period to the epidemic normality.

**Fig 6:**
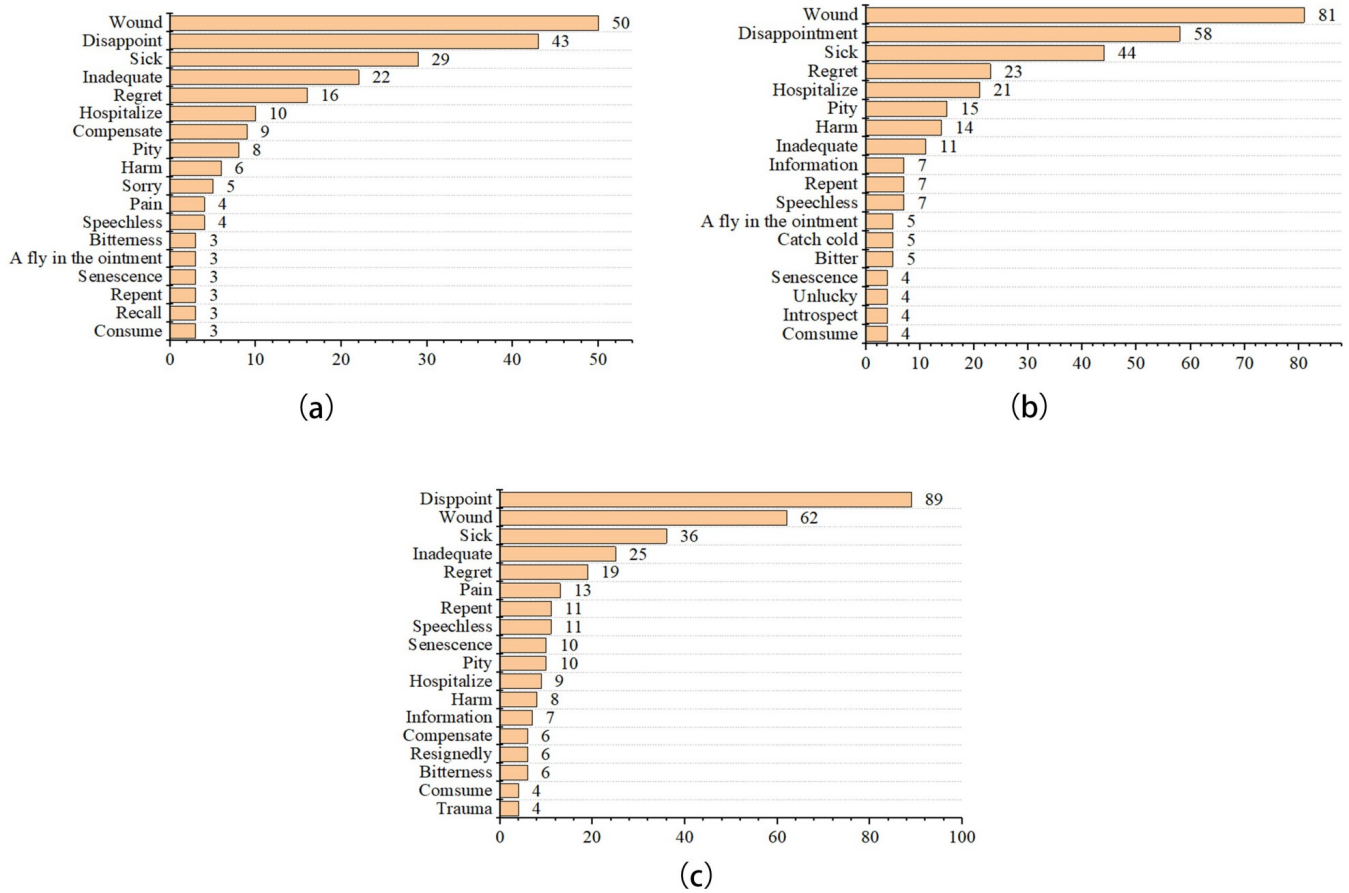
Emotional high-frequency words of sadness in the pre-COVID-19 (a), COVID-19 lockdown (b), and normalization era (c).

As shown in Fig 6, the top words of sadness emotion in the health product reviews were “wound”, “sick”, and “disappoint” in three phases. After the outbreak of COVID-19, consumers expressed “wound” more often. But the number of times that “wound” was expressed decreased significantly after the normalization of COVID-19 prevention and control. “Disappoint” changed similarly. “Sick” has been expressed as increased and then decreased as COVID-19 progresses.

**Fig 7:**
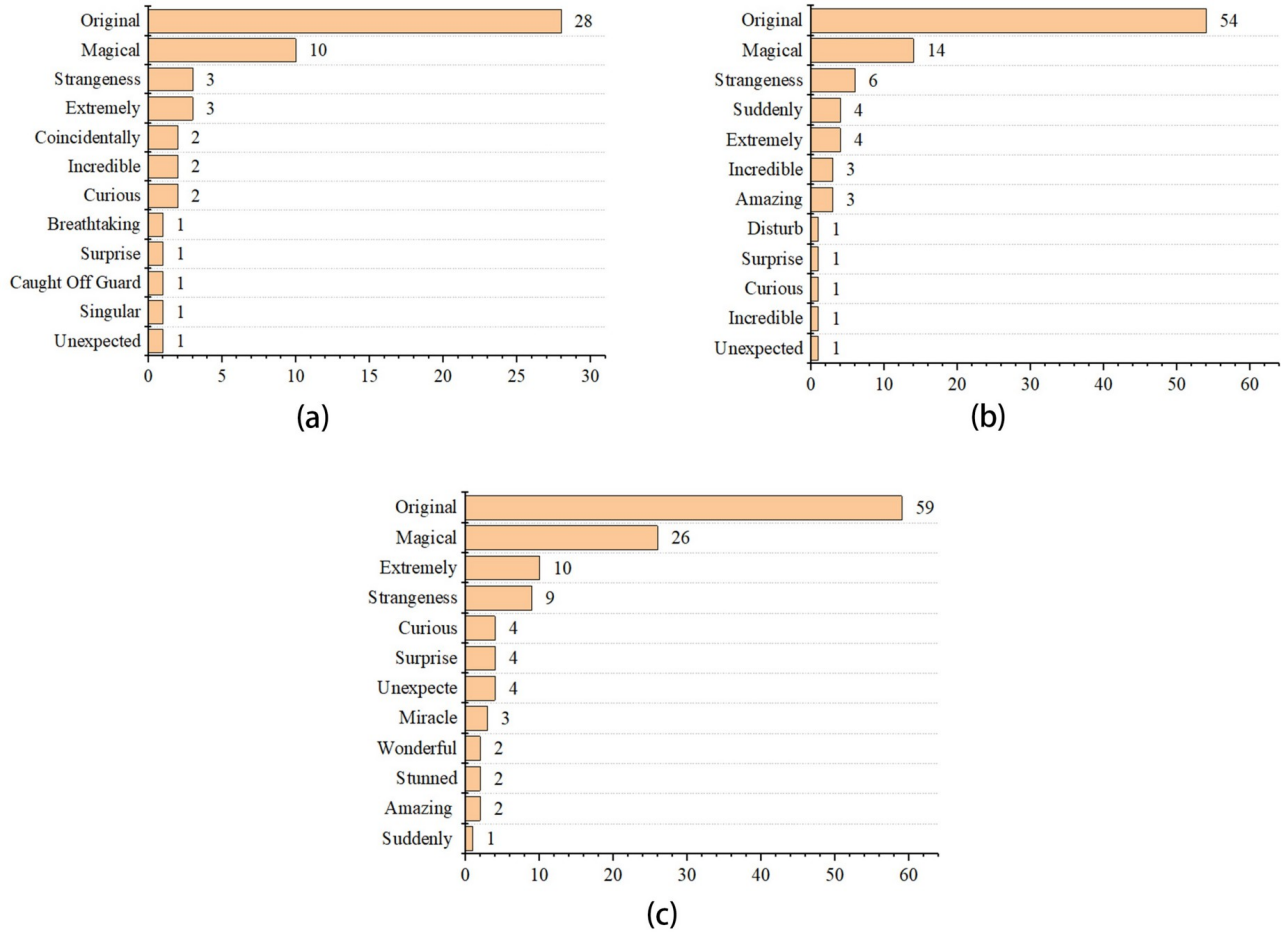
Emotional high-frequency words of surprise in the pre-COVID-19 (a), COVID-19 lockdown (b), and normalization era (c).

For surprise emotion, consumers expressed words like “original” and “magical” more often in three periods, followed by “suddenly”, “strangeness”, and “extremely”. Among them, “original” and “magical” increased twice during COVID-19 normalization compared to before the COVID-19 period. “Strangeness” and “extremely” have also risen remarkably after the COVID-19 outbreak. It can also be seen that “amazing” only appears during COVID-19 in Fig 7.

**Fig 8:**
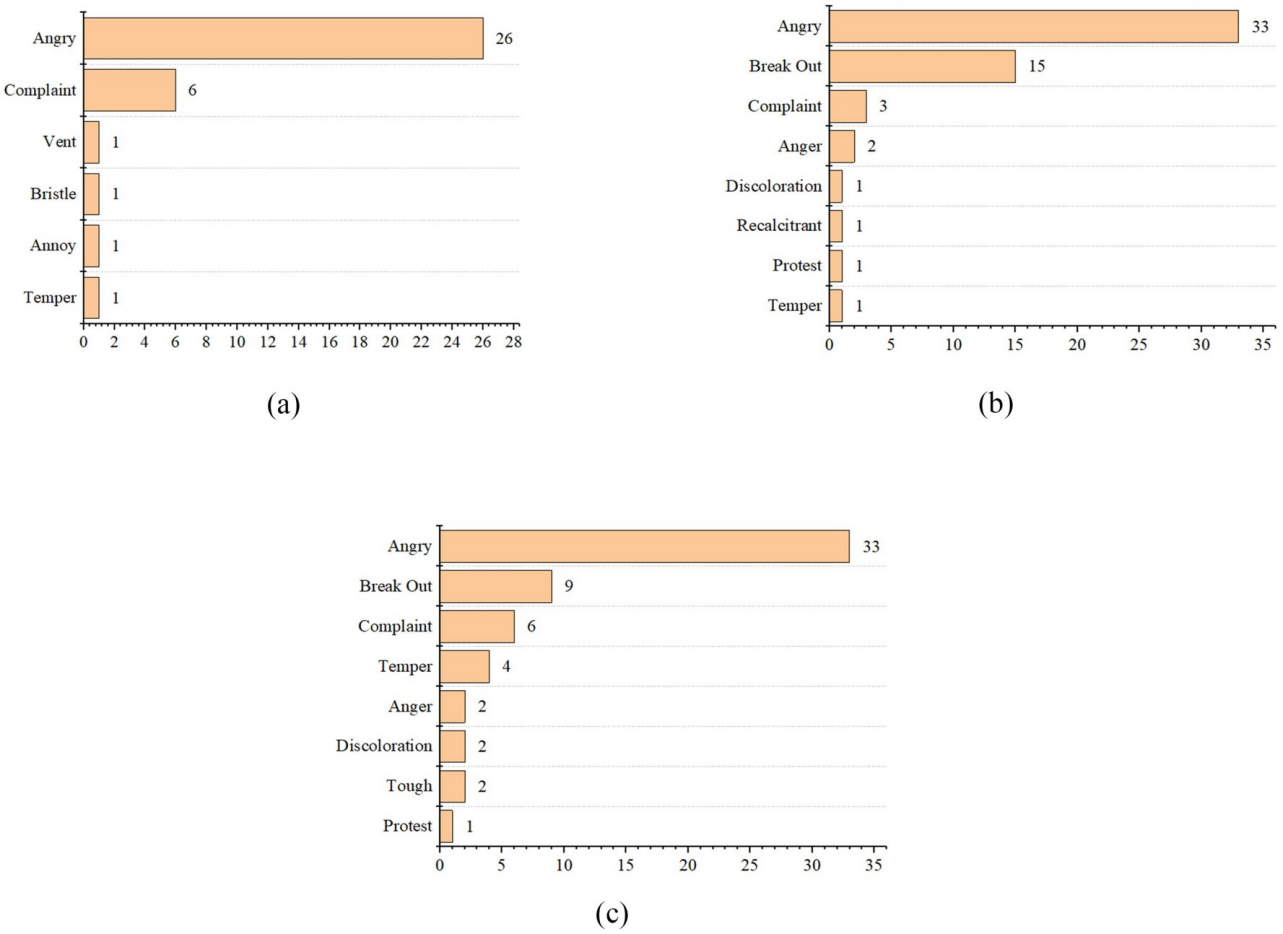
Emotional high-frequency words of anger in the pre-COVID-19 (a), COVID-19 lockdown (b), and normalization era (c).

“Angry” was expressed frequently. “Complaint” was the secondary frequent word in pre-COVID-19. “Break out” was the frequent secondary word in the two other phases. By comparing Fig 8, we can see that “angry” did not increase much before and during COVID-19. “Break out” spiked during the epidemic, but the number of times consumers expressed it dropped after the epidemic.

### 4.3 Emotion bias in the context of COVID-19

Emotion is the study’s dependent variable, classified into seven categories: liking, happy, sorrow, anger, fear, disgust, and surprise, in no particular order.

As a result, for analysis, we chose an unordered multiple logistic regression model. The multiple logistic regression model compares the selected category and the reference category in each variable to examine the influence of each element, and the result reflects the event’s probability. The formula is as follows:

$$logit\left(P_{i}=ln\frac{P_{i}}{1-P_{i}}\right)=\beta_{0}+\Sigma_{i=1}^{n}\beta_{i}x_{i}$$ (4)

The reference category is denoted by *i*. *Pi* is the conditional probability of the *j* − *th* event occurring. *βi* is the regression coefficient of the independent variable, *xi* is the independent variable, and *βo* represents a constant term.

The result of data analysis using logistics regression (Table 2) explained that time was a significant factor to fear emotion (p-value = 0.001<0.05), sadness emotion (p-value = 0.042<0.05) and like emotion (p-value = 0.032<0.05). While time was not a significant factor in other emotions(p>0.05). The COVID-19 normalization period is the reference object. In the fear emotion, compared with the COVID-19 period and the COVID-19 normalization period, the influence of time played an important role in expressing fear emotion, and the impact was positive. Combined with adjusted OR, the OR value of the COVID-19 era was 0.669 times that of the COVID-19 normalization era, which showed that the probability of the COVID-19 era expressing anger is 0.669 times that of the COVID-19 normalization era. Similarly, this variable had a significantly positive impact on like emotion (β = 0.17) and sadness emotion(β = 0.052).

**Table 2:** Emotion bias across time and product categories.

Dependent variable	Independent variables	β	P-value	Adjusted OR	95\%C.I of adjusted OR
Anger	The pre-COVID-19 period	0.089	0.676	1.093	(0.719,1.662)
	The COVID-19 lockdown period	0.145	0.439	1.156	(0.801,1.667)
	Traditional Chinese and Western medicines	1.432	0	4.188	(2.644,6.633)
	Nutritional products	0.401	0.07	1.494	(0.967,2.306)
	Functional foods	1.233	0	3.433	(2.036,5.789)
Disgust	The pre-COVID-19 period	0.008	0.847	1.008	(0.929,1.094)
	The COVID-19 lockdown period	-0.006	0.869	0.994	(0.926,1.067)
	Traditional Chinese and Western medicines	0.459	0	1.582	(1.423,1.758)
	Nutritional products	0.482	0	1.619	(1.505,1.741)
	Functional foods	0.453	0	1.573	(1.399,1.769)
Fear	The pre-COVID-19 period	-0.145	0.245	0.865	(0.676,1.105)
	The COVID-19 lockdown period	0.290	0.001	1.337	(1.118,1.599)
	Traditional Chinese and Western medicines	0.489	0	1.63	(1.315,2.022)
	Nutritional products	-0.715	0	0.489	(0.399,0.601)
	Functional foods	-0.594	0.001	0.552	(0.384,0.975)
Like	The pre-COVID-19 period	0.170	0.032	1.185	(1.014,1.384)
	The COVID-19 lockdown period	0.090	0.197	1.094	(0.955,1.253)
	Traditional Chinese and Western medicines	0.217	0.028	1.243	(1.024,1.509)
	Nutritional products	0.045	0.522	1.046	(0.911,1.201)
	Functional foods	0.313	0.004	1.367	(1.108,1.686)
Happiness	The pre-COVID-19 period	-0.145	0.374	0.865	(0.629,1.191)
	The COVID-19 lockdown period	-0.202	0.145	0.817	(0.622,1.073)
	Traditional Chinese and Western medicines	0.173	0.379	1.189	(0.809,1.747)
	Nutritional products	-0.018	0.897	0.982	(0.748,1.289)
	Functional foods	-0.170	0.492	0.844	(0.520,1.370)
Sadness	The pre-COVID-19 period	-0.011	0.709	0.989	(0.932,1.049)
	The COVID-19 lockdown period d	0.052	0.042	1.054	(1.002,1.108)
	Traditional Chinese and Western medicines	0.140	0	1.15	(1.066,1.241)
	Nutritional products	0.028	0.284	1.028	(0.977,1.081)
	Functional foods	0.594	0	1.81	(1.667,1.967)

Regarding the product categories, healthcare equipment was the reference object. This variable had a significant impact on anger emotion, disgust emotion, fear emotion, like emotion, and sadness emotion (p-value <0.05). In sad emotion, the value of the coefficient for traditional Chinese and Western medicines increases by 0.140 units compared to healthcare equipment. In other words, it positively affected the emotional expression of sadness emotion. Similarly, functional foods had a significantly positive impact on emotional expression (β = 0.594). Consumers purchasing traditional Chinese and Western medicines and functional foods were more likely than consumers purchasing healthcare equipment to express sadness emotion.

Traditional Chinese and Western medicines and functional foods played an essential role in the emotional expression of like (p-value <0.05), and the impact was positive. The probability of preferring expressing happiness emotion by consumers purchasing healthcare equipment was 24.3% lower than consumers purchasing traditional Chinese and Western medicines (OR = 1.243) and 36.7% lower than consumers purchasing functional foods (OR = 1.367). Nutritional products failed the significance test.

For fear emotion, traditional Chinese and Western medicines were positively correlated with the emotional expression of fear, and the impact was significant (p-value = 0.00<0.05). Nutritional products (p = 0.00<0.05) and functional foods (p = 0.001<0.05) had a very significant effect on expressing fear, and the relationship was negative. With the other conditions unchanged, the rate of occurrence of expressing fear emotion increased by 1.63 times for traditional Chinese and Western medicines (OR = 1.63). The rate of occurrence of expressing fear emotion decreased by 0.489 times for nutritional products (OR = 0.489) and decreased by 0.552 times for functional foods (OR = 0.552).

In anger and disgust emotion, product categories were significantly positive in the multinomial ordinal logistic regression model (p-value<0.05). Consumers who purchased traditional Chinese and Western medicines, functional foods and nutritional products were more inclined to express anger and disgust emotion.

The results indicated that product categories and time strongly related to seven emotional expressions.

### 4.4 Bias of emotional intensity in the context of COVID-19

In question 3, the dependent variable, emotional intensity, was a continuous variable, and the independent variable was categorical. One-way analysis of variance was performed to analyze consumers’ emotional intensity across time and product categories.

Table 3 revealed that there was a significant difference in time and anger emotion (F = 4.819, P-value = 0.038<0.05). It meant the time significantly affected the intensity of anger emotion. There was, however, no significant impact between time and other emotions (p-value>0.05).

**Table 3:** One-way analysis of variance between time and product categories and emotional intensity.

	Sum of square	Df	Mean square	F	p-value
Happiness					
Time	0.790	2.000	0.395	0.391	0.687
Product categories	7.213	3.000	2.404	7.233	0.011
Like					
Time	1.160	2.000	0.580	0.599	0.570
Product categories	7.291	3.000	2.430	7.532	0.010
Anger					
Time	4.823	2.000	2.411	4.819	0.038
Product categories	1.175	3.000	0.392	0.384	0.767
Sadness					
Time	1.478	2.000	0.739	0.792	0.482
Product categories	1.563	3.000	0.521	0.502	0.692
Fear					
Time	2.611	2.000	1.305	1.618	0.251
Product categories	1.288	3.000	0.429	0.400	0.757
Disgust					
Time	2.373	2.000	1.187	1.424	0.290
Product categories	5.819	3.000	1.940	3.829	0.048
Surprise					
Time	0.613	2.000	0.307	0.313	0.739
Product categories	1.486	3.000	0.495	0.498	0.694

The p-value of product categories in sadness, fear, anger, and surprise did not reach the corrected significance level. Therefore, the result showed no significant difference in the intensity of the five emotional expressions. Product categories were statistically significant in happiness, like and disgust emotions (P-value<0.05), and F-value was high. The intensity of emotional expression differed significantly among the three emotions.

One-way ANOVA can only test whether the control variables significantly affected the observed variables. It cannot test the extent to which the control variables affect the observed variables at different levels. Therefore, it was necessary to use post-hoc multiple testing methods to test the degree of influence of control variables on test variables at different levels.

The time multiple comparison test results are shown in Table 4.

**Table 4:** Multiple comparison tests of time.

(I)Time	(J)Time	(I-J)Mean Difference
		Anger
The pre-COVID-19 period	The COVID-19 lockdown period	0.145613903
	The COVID-19 normalization period	1.4116915*
The COVID-19 lockdown period	The pre-COVID-19 period	-0.145613903
	The COVID-19 normalization period	1.2660776*
The COVID-19 normalization period	The pre-COVID-19 period	-1.4116915*
	The COVID-19 lockdown period	-1.2660776*

* represents p-value<0.05.

The Bonferroni post hoc test has examined multiple comparisons [51]. The post hoc tests using the Bonferroni correction revealed that the intensity of consumers’ anger expression during the pre-COVID-19 and COVID-19 lockdown was significantly higher than during the COVID-19 normalization period.

The results of the multiple comparison test for product categories were shown in Table 5.

**Table 5:** Multiple comparison tests of product categories.

(I)Product categories	(J)Product categories	(I-J)Mean Difference		
		Happiness	Like	Disgust
Traditional Chinese and Western medicines	Nutritional products	-1.09315*	-1.65697*	1.32822
	Functional foods	0.403911	-1.0014	-0.5945
	Healthcare equipment	-1.49706*	-2.06236*	0.199012
Nutritional products	Traditional Chinese and Western medicines	1.09315*	1.65697*	-1.32823
	Functional foods	1.49706*	0.65549	-1.92276*
	Healthcare equipment	-0.403911	-0.4053	-1.12921
Functional foods	Traditional Chinese and Western medicines	-0.403911	1.0014	0.594536
	Nutritional products	-1.49706*	-0.6554	1.9227624*
	Healthcare equipment	-1.90097*	-1.0608	0.793548
Healthcare equipment	Traditional Chinese and Western medicines	1.49706*	2.06236*	-0.19901
	Nutritional products	0.403911	0.40539	1.129214
	Functional foods	1.900978*	1.06088	-0.79355

* represents p-value<0.05.

As can be seen from the mean difference in Table 5, the emotional intensity of nutritional products and healthcare equipment reviews was higher than in traditional Chinese and Western medicine and functional foods when consumers expressed happiness emotions. When consumers expressed like emotion, the emotional intensity of nutritional products and healthcare equipment reviews was higher, and the emotional intensity of traditional Chinese and Western medicine reviews was lower. When consumers expressed disgust emotions, the emotional intensity of functional foods comments was higher, while the emotional intensity of nutritional products was lower.

### 4.5 Discussion

In this study, we track the emotion bias from November 11, 2019, to July 1, 2020, covering the pre-COVID-19 period, the COVID-19 lockdown period, and the COVID-19 normalization period. Specifically, this paper uses the DUTIR Emotion Ontology set. Firstly, emotion is divided into seven categories to analyze consumer emotion. Then this research analyzes emotion bias and emotional intensity bias using statistical methods. A minimum of three significant findings emerge from this study.

Firstly, this paper counts the high-frequency words of seven emotions during three periods. “Outbreak” and “getting angry” become high-frequency words for anger emotion during the epidemic, which is associated with the disease’s origin in Wuhan, China. A study [52] showed that fear is the predominant negative reaction to COVID-19. Among them, “virus” is the most negative word for fear emotion, followed by “intensely” and “urgent”. With the changes in the spread of infectious diseases, people would pay attention to and search for virus-related vocabulary [53]. The sense of oppression caused by the virus is getting heavier, and consumers would seek ways to avoid infection. Consumers’ demand for health increases. As a primitive sensation, disgust was associated with a propensity to avoid other people and diseases [54]. Therefore, the number of consumers expressing "blame" increases significantly in the COVID-19 lockdown and normalization phase and is twice that before the epidemic.

As COVID-19 progress, however, consumer emotion tends to become more optimistic as essential goods and medical supplies are not in short supply, which can meet the health needs of consumers. There is not much difference in the expression of happiness and like emotion during the three periods. This is because JD.com product reviews are mostly positive reviews nowadays. Sellers hope that more positive reviews could attract consumers to increase their turnover so they will delete negative product reviews. “Regret”, “sick”, “disappoint”, and “wound” had become high-frequency words for sadness emotion in the pre-and amid-COVID-19 phases. With so much uncertainty and mental trauma caused by COVID-19, people are increasingly worried about their health, loved ones, and economic future. Vaccines are the primary factor in the fight against neo-coronavirus and are the ideal solution to COVID-19 [9]. Currently, the Chinese government is promoting vaccination against New Coronavirus through various social media, and vaccination rates have reached over 90%.

Secondly, there are differences in the expression of emotion types among consumers in different periods. Compared to the COVID-19 normalization phase, consumers have more willingness to express fear and sadness during the epidemic lockdown phase and have more willingness to express like emotions before COVID-19. There is no significant difference in other emotional expression in the three periods. This finding was in line with prior research, which found that negative emotions like anger and sadness are more prevalent during pandemics [55]. The continuing Covid-19 poses a global threat to human life and has evoked an array of negative emotions, including fear, rage, and despair. And consumers’ life satisfaction declines [9]. Some studies have shown that life satisfaction and health correlate positively [56]. As a result, people are more willing to focus on features and behaviors that can help them regain control and assurance, such as buying, to lessen unpleasant sensations [57]. Changes in consumer behavior might be explained as a corrective response to the COVID-19 emergency’s dread and worry.

Besides, there are differences in the expression of emotion types among consumers in different product categories. Consumers who purchased traditional Chinese and Western medicine, nutritional products, and functional foods are more willing to express anger and disgust than those who purchased healthcare equipment. Consumers who purchase traditional Chinese and Western medicine and healthcare equipment are more willing to express fear emotion. And consumers who purchase traditional Chinese and Western medicine and functional foods are more willing to express like and sadness emotions. To sum up, consumers who purchase traditional Chinese and Western medicine, nutritional products, and functional foods are more willing to express their emotions. COVID-19 has persisted significantly longer than previous epidemics. Due to fears about infection, online shopping has risen rapidly, and people are hoarding health supplements like nutritional products and functional foods for fear of the uncertainty brought on by the disaster. After that, consumers will share their experiences on the platform JD to relieve their worries and bring some help to others. A diet characterized by healthy foods reduces the risk and severity of COVID-19 [9]. Correspondingly, healthcare equipment is a high-priced product that is beyond the reach of consumers. For the average consumer, healthcare equipment is used less frequently and must be used under a relevant professional’s guidance. And the number of people who consume it is less, so fewer people are expressing emotions.

Finally, time significantly differed in the emotional intensity of expressing anger. The emotional intensity of product reviews in the pre-COVID-19 and the lockdown phase was higher, and the emotional intensity in the normalization phase was lower. Anger is viewed as a negative emotion with a high level of arousal. And anger intensity was high during COVID-19. One reason is that the significant fear signals identified here are red lights regarding escalating consumer tension, which could lead to psychological severe suffering due to diminished self-control [58]. In this circumstance, rage emotions would be more intense. Wuhan’s closure of the city for nearly three months, with consumers unable to leave their homes and unable to secure basic necessities and inadequate supplies of epidemic prevention supplies, was a significant reason for the change in consumer sentiment.

Reviews of different product types have differences in the expression intensity of happiness, like, disgust emotion. The emotional intensity of nutritional products and healthcare equipment reviews is higher in happiness and like emotion. Nutritional products and healthcare equipment can regulate body functions and increase immunity. Positive sensations of relaxation and happiness were experienced more frequently and powerfully. When consumers use Nutritional products and healthcare equipment, consumers naturally become happy when they feel protected. Yet, the intensity of disgust emotion in functional food reviews is higher when consumers are expressing disgust. Some studies show consumers dread functional foods significantly more than organic and conventional food [59]. Psychology explains that high levels of fear produce awe and obedience. Low levels of fear produce aversion. Fear can lead to aversion. This also indirectly reflects that the findings of this thesis are well-founded. As a result, this may cause an aversion to functional foods among consumers.

## 5 Conclusion

Changes in customer behavior have had a substantial impact on the health product business as the COVID-19 influence. Regarding this research, we collect health product reviews in JD mall from November 11, 2019, to January 22, 2020, in China. This study examines emotion bias in the three stages (the pre-COVID-19 era, the COVID-19 lockdown era, and the COVID-19 normalization era). The research result indicates that “outbreak” and “get angry” become high-frequency words for anger emotion during the epidemic, and “virus” is the most negative word for fear emotion. “Regret”, “sick”, and “the wound” become high-frequency words for sadness emotion amid the COVID-19 lockdown. However, there are not many differences in happiness and like emotions before and during COVID-19. Further, the study reveals that time and product categories have a difference in the expression of the following emotions: fear, sadness, and like. Besides, product categories have a very significant effect on expressing disgust and anger. Finally, the time has a difference in the emotional intensity of anger, and product categories have a significant difference in the emotional intensity of disgust, like, and happiness. The current research has substantial implications theoretically and practically by revealing changes in consumer emotion that lead to consumer behavioral choices during COVID-19.

Our research has three major implications for a health product company. First, COVID-19 is projected to have a long-term impact on consumer behavior because the recovery will not be a quick process. The fear of the coronavirus is still present. As a result, marketers can actively advertise how a company employs sanitary habits and safety standards to assuage consumers’ fears about the threat, thus establishing a healthy and honest brand image.

Secondly, industry-wide campaigns and consumer communication can be beneficial by emphasizing the low danger of viral transmission through food handling and consumption. Enterprises can develop a system to better communicate with dealers and consumers, transmit their information and declare their commitment to consumers, and improve consumers’ trust in the brand.

Finally, since consumers who purchase nutritional products and healthcare equipment will express like and happiness emotions more often, companies can focus on the research and development of these two types of health products and develop strong advertising campaigns that emphasize the professionalism of the enterprise and the reliability of health products.

While our study contributes to theory and practices, there are some limitations, too. First, this paper’s selection of review websites is limited. The fact that only JD mall is chosen could lead to a platform bias. As a result, future research can also look at COVID-19’s impact on different platforms. Second, this study only considers time and product categories in selecting variables. In future research, more variables (age, gender, etc.) can be selected to explore their role in emotional expression in more dimensions. Finally, the current paper focused on consumer sentiment changes over some time. However, as the global economy enters a phase of recovery, consumer behavior may periodically change, depending on the pandemic’s development and individuals’ ability to adjust to the ever-changing "new normality." As a result, it is advisable to do a year-by-year analysis over the next several years to acquire a more in-depth insight into the post-pandemic consumer. Hence, dealing with these limitations could be the direction for future research.

## References

[1] Weiss S R, Navas-Martin S. Coronavirus pathogenesis and the emerging pathogen severe acute respiratory syndrome coronavirus[J]. Microbiology and molecular biology reviews, 2005, 69(4): 635–664. 16339739 10.1128/MMBR.69.4.635-664.2005 PMC1306801

[2] Umakanthan S, Sahu P, Ranade A V, et al. Origin, transmission, diagnosis and management of coronavirus disease 2019 (COVID-19)[J]. Postgraduate medical journal, 2020, 96(1142): 753–758. 32563999 10.1136/postgradmedj-2020-138234 PMC10016932

[3] Umakanthan S, Bukelo M M, Gajula S S. The Commonwealth Caribbean COVID-19: Regions Resilient Pathway During Pandemic[J]. Frontiers in Public Health, 2022, 10: 844333. 35664108 10.3389/fpubh.2022.844333 PMC9160791

[4] World Health Organization. WHO Director-General’s Opening Remarks at the Media Briefing on COVID-19–11 March 2020. https://www.who.int/dg/speeches/detail/who-director-general-s-opening-remarks-at-the-media-briefing-on-covid-19---11-march-2020 (accessed on 17 June 2020).

[5] Attwood S.; Hajat C. How will the COVID-19 pandemic shape the future of meat consumption? Public Health Nutr. 2020, 23, 3116–3120. doi: 10.1017/S136898002000316X 32782062 PMC7533480

[6] Allington D.; Duffy B.; Wessely S.; Dhavan N.; Rubin J. Health-protective behaviour, social media usage and conspiracy belief during the COVID-19 public health emergency. Psychol. Med. 2020, 9, 1–7. doi: 10.1017/S003329172000224X 32513320 PMC7298098

[7] Wichaidit W.; Naknual S.; Kleangkert N.; Liabsuetrakul T. Installation of pedal-operated alcohol gel dispensers with behavioral nudges and changes in hand hygiene behaviors during the COVID-19 pandemic: A hospital-based quasi-experimental study. J. Public Health Res. 2020, 9, 1863. doi: 10.4081/jphr.2020.1863 33150146 PMC7607228

[8] Boons, F.; Browne, A.; Burgess, M.; Ehgartner, U.; Hirth, S.; Hodson, M. et al. Covid-19, Changing Social Practices and the Transition to Sustainable Production and Consumption; Version 1.0 (May 2020); Sustainable Consumption Institute: Manchester, UK, 2020.

[9] Umakanthan S, Patil S, Subramaniam N, et al. COVID-19 vaccine hesitancy and resistance in India explored through a population-based longitudinal survey[J]. Vaccines, 2021, 9(10): 1064. 34696172 10.3390/vaccines9101064 PMC8537475

[10] Jiao W Y, Wang L N, Liu J, et al. Behavioral and emotional disorders in children during the COVID-19 epidemic[J]. The Journal of pediatrics, 2020, 221: 264. 32248989 10.1016/j.jpeds.2020.03.013 PMC7127630

[11] Zhong Y, Oh S, Moon H C. What can drive consumers’ dining-out behavior in China and Korea during the COVID-19 pandemic?[J]. Sustainability, 2021, 13(4): 1724.

[12] Omar N A, Nazri M A, Ali M H, et al. The panic buying behavior of consumers during the COVID-19 pandemic: Examining the influences of uncertainty, perceptions of severity, perceptions of scarcity, and anxiety[J]. Journal of Retailing and Consumer Services, 2021, 62: 102600.

[13] Di Crosta A, Ceccato I, Marchetti D, et al. Psychological factors and consumer behavior during the COVID-19 pandemic[J]. PloS one, 2021, 16(8): e0256095. 34398916 10.1371/journal.pone.0256095 PMC8366984

[14] Du H, Yang J, King R B, et al. COVID-19 increases online searches for emotional and health-related terms[J]. Applied Psychology: Health and Well-Being, 2020, 12(4): 1039–1053. 33052612 10.1111/aphw.12237 PMC7675240

[15] Twenge J M, Joiner T E. Mental distress among US adults during the COVID-19 pandemic[J]. Journal of Clinical Psychology, 2020, 76(12): 2170–2182. 33037608 10.1002/jclp.23064 PMC7675251

[16] Ling M., Kothe E.J., & Mullan B.A. (2019). Predicting intention to receive a seasonal influenza vaccination using Protection Motivation Theory. Social Science & Medicine, 233, 87–92. doi: 10.1016/j.socscimed.2019.06.002 31195194

[17] Wang M Y, Zhang P Z, Zhou C Y, et al. Effect of emotion, expectation, and privacy on purchase intention in WeChat health product consumption: The mediating role of trust[J]. International Journal of Environmental Research and Public Health, 2019, 16(20): 3861. 31614749 10.3390/ijerph16203861 PMC6843468

[18] Harba J N, Tigu G, Davidescu A A M. Exploring consumer emotions in pre-pandemic and pandemic times. A sentiment analysis of perceptions in the fine-dining restaurant industry in Bucharest, Romania[J]. International Journal of Environmental Research and Public Health, 2021, 18(24): 13300. 34948908 10.3390/ijerph182413300 PMC8704477

[19] Luneva E.E., Banokin P.I., Yefremov A.A. and Tiropanis T., "Method of evaluation of social network user sentiments based on fuzzy logic", Key Engineering Materials, vol. 685, pp. 847–851, Feb. 2016.

[20] Boon-Itt S, Skunkan Y. Public perception of the COVID-19 pandemic on Twitter: Sentiment analysis and topic modeling study[J]. JMIR Public Health and Surveillance, 2020, 6(4): e21978. 33108310 10.2196/21978 PMC7661106

[21] Lwin M O, Lu J, Sheldenkar A, et al. Global sentiments surrounding the COVID-19 pandemic on Twitter: analysis of Twitter trends[J]. JMIR public health and surveillance, 2020, 6(2): e19447. 32412418 10.2196/19447 PMC7247466

[22] Wang T, Lu K, Chow K P, et al. COVID-19 sensing: negative sentiment analysis on social media in China via BERT model[J]. Ieee Access, 2020, 8: 138162–138169. 34812342 10.1109/ACCESS.2020.3012595 PMC8545339

[23] Smith K S, McCreadie R, Macdonald C, et al. Regional sentiment bias in social media reporting during crises[J]. Information Systems Frontiers, 2018, 20(5): 1013–1025.

[24] Muzi S, Sansò A, Pace C S. What’s Happened to Italian Adolescents During the COVID-19 Pandemic? A Preliminary Study on Symptoms, Problematic Social Media Usage, and Attachment: Relationships and Differences With Pre-pandemic Peers[J]. Frontiers in Psychiatry, 2021, 12. 10.3389/fpsyt.2021.590543 PMC8110826 33986698

[25] S. Meyer. Understanding the Covid-19 effect on online shopping behaviour, BigCommerce. https://www.bigcommerce.com/blog/covid-19ecommerce/#conclusion (2020), Accessed 5th Jul 2020

[26] Laato S, Islam A K M N, Farooq A, et al. Unusual purchasing behavior during the early stages of the COVID-19 pandemic: The stimulus-organism-response approach[J]. Journal of Retailing and Consumer Services, 2020, 57: 102224.

[27] Kirk C P, Rifkin L S. I’ll trade you diamonds for toilet paper: Consumer reacting, coping and adapting behaviors in the COVID-19 pandemic[J]. Journal of business research, 2020, 117: 124–131. 32834208 10.1016/j.jbusres.2020.05.028 PMC7241317

[28] Yıldırım M, Akgül Ö, Geçer E. The effect of COVID-19 anxiety on general health: The role of COVID-19 coping[J]. International Journal of Mental Health and Addiction, 2022, 20(2): 1110–1121. 33456406 10.1007/s11469-020-00429-3 PMC7799156

[29] Tröster B, Küblböck K. Unprecedented but not unpredictable: Effects of the COVID-19 crisis on commodity-dependent countries[J]. The European Journal of Development Research, 2020, 32(5): 1430–1449. 33100597 10.1057/s41287-020-00313-9 PMC7575855

[30] Valaskova K, Durana P, Adamko P. Changes in consumers’ purchase patterns as a consequence of the COVID-19 pandemic[J]. Mathematics, 2021, 9(15): 1788.

[31] Li S, Wang Y, Xue J, et al. The impact of COVID-19 epidemic declaration on psychological consequences: a study on active Weibo users[J]. International journal of environmental research and public health, 2020, 17(6): 2032. 32204411 10.3390/ijerph17062032 PMC7143846

[32] Kiecolt-Glaser J K, McGuire L, Robles T F, et al. Emotions, morbidity, and mortality: New perspectives from psychoneuroimmunology[J]. Annual review of psychology, 2002, 53(1): 83–107. 10.1146/annurev.psych.53.100901.135217 11752480

[33] Yoo S Y, Song J I, Jeong O R. Social media contents based sentiment analysis and prediction system[J]. Expert Systems with Applications, 2018, 105: 102–111.

[34] Liu B, Hu M, Cheng J. Opinion observer: analyzing and comparing opinions on the web[C]//Proceedings of the 14th international conference on World Wide Web. 2005: 342–351.

[35] Chen J M, Lin H F, Yang Z H. Automatic acquisition of emotional vocabulary based on syntax[J]. CAAI Transactions on Intelligent Systems, 2009, 4(2): 100–106.

[36] Li A P, Di P, Duan G L. Document sentiment orientation analysis based on sentence weighted algorithm[J]. Journal of Chinese Computer Systems, 2015, 36(10): 2252–2256.

[37] Yu S, Eisenman D, Han Z. Temporal dynamics of public emotions during the COVID-19 pandemic at the epicenter of the outbreak: sentiment analysis of Weibo posts from Wuhan[J]. Journal of medical Internet research, 2021, 23(3): e27078. 33661755 10.2196/27078 PMC7977613

[38] Manek A.S., Shenoy P.D., Mohan M.C., et al. Aspect term extraction for sentiment analysis in large movie reviews using gini index feature selection method and SVM classifier. World Wide Web-internet & Web Information Systems, 20 (2) (2017), pp. 135–154

[39] Rout J.K., Choo K.K.R., Dash A.K., et al. A model for sentiment and emotion analysis of unstructured social media text. Electron. Commer. Res. (2017), pp. 1–19

[40] Abbasi A., Chen H., Salem A. Sentiment analysis in multiple languages. ACM Trans. Inf. Syst., 26 (3) (2008), pp. 1–34

[41] Samuel J, Ali G G, Rahman M, et al. Covid-19 public sentiment insights and machine learning for tweets classification[J]. Information, 2020, 11(6): 314.

[42] Xu L. Constructing the affective lexicon ontology. J China Soc Entific Tech Inf 2008;27(2):180–185.

[43] Iglesias-Sánchez PP, Vaccaro Witt GF, Cabrera FE, Jambrino-Maldonado C. The contagion of sentiments during the COVID-19 pandemic crisis: The case of isolation in Spain. Int J Environ Res Public Health 2020 Aug 14;17(16):5918 doi: 10.3390/ijerph17165918 32824110 PMC7460470

[44] Lucka N S, Caldieraro F, Zanini M T. The influence of gender stereotyping and issue advocacy on consumer sentiment[J]. Marketing Intelligence & Planning, 2021.

[45] Aslam N. Attachment styles as a predictor of emotional expression among depressed and non depressed individuals[J]. Journal of Behavioural Sciences, 2013, 23(1): 102.

[46] Boshoff C, Van Eeden S M. South African consumer sentiment towards marketing: A longitudinal analysis[J]. South African Journal of Business Management, 2001, 32(2): 23–33.

[47] Acevedo D N, Fogleman C, Daniel Ura J. Peasants and bankers: Education, consumer sentiment, and presidential approval[J]. Presidential Studies Quarterly, 2017, 47(2): 230–244.

[48] Liu Y, Bi J W, Fan Z P. Ranking products through online reviews: A method based on sentiment analysis technique and intuitionistic fuzzy set theory[J]. Information Fusion, 2017, 36: 149–161.

[49] Zhang W, Kong S, Zhu Y, et al. Sentiment classification and computing for online reviews by a hybrid SVM and LSA based approach[J]. Cluster Computing, 2019, 22(5): 12619–12632.

[50] Al-Natour S, Turetken O. A comparative assessment of sentiment analysis and star ratings for consumer reviews[J]. International Journal of Information Management, 2020, 54: 102132.

[51] Devereux E, Grimmer L, Grimmer M. Consumer engagement on social media: Evidence from small retailers[J]. Journal of Consumer Behaviour, 2020, 19(2): 151–159.

[52] Sesagiri Raamkumar A, Tan S, Wee H. Measuring the Outreach Efforts of Public Health Authorities and the Public Response on Facebook During the COVID-19 Pandemic in Early 2020: Cross-Country Comparison. J Med Internet Res 2020 May 19;22(5):e19334 doi: 10.2196/19334 32401219 PMC7238862

[53] Reynolds D, Garay J, Deamond S, Moran M, Gold W, Styra R. Understanding, compliance and psychological impact of the SARS quarantine experience. Epidemiol Infect 2008 Jul;136(7):997–1007. doi: 10.1017/S0950268807009156 17662167 PMC2870884

[54] Trzebiński W, Baran R, Marciniak B. Did the COVID-19 Pandemic Make Consumers Shop Alone? The Role of Emotions and Interdependent Self-Construal[J]. Sustainability, 2021, 13(11): 6361.

[55] Mota N B, Weissheimer J, Ribeiro M, et al. Dreaming during the Covid-19 pandemic: Computational assessment of dream reports reveals mental suffering related to fear of contagion[J]. PloS one, 2020, 15(11): e0242903. 33253274 10.1371/journal.pone.0242903 PMC7703999

[56] Palmore E, Luikart C. Health and social factors related to life satisfaction[J]. Journal of health and social behavior, 1972: 68–80. 5025756

[57] Wilkens J, San Diego Union-Tribune. Psychologists say why we hoard: Fear at root of panic-buying [J]. San Diego Union-Tribune [Internet], 2020, 22.

[58] Moroń M, Biolik-Moroń M. Trait emotional intelligence and emotional experiences during the COVID-19 pandemic outbreak in Poland: A daily diary study[J]. Personality and Individual Differences, 2021, 168: 110348. 32843781 10.1016/j.paid.2020.110348 PMC7439821

[59] Laros F J M, Steenkamp J B E M. Emotions in consumer behavior: a hierarchical approach[J]. Journal of business Research, 2005, 58(10): 1437–1445.

